# Supplementary material for: A lipocalin and a Hedgehog-related protein are partners in the Caenorhabditis elegans precuticle apical extracellular matrix
Source: Genetics. 2026 Aug 19;234(3):iyag218. doi: 10.1093/genetics/iyag218 (PMC13732703; doi:10.1093/genetics/iyag218)
Supplement: Supplementary file 1 [file iyag218_supplementary_data.zip › Peer_Review_History_309741R1.pdf]

GENETICS

A Lipocalin and a Hedgehog-related protein are partners in the C. elegans pre-cuticle apical extracellular matrix

Nicholas Serra, Jason Chen, Susanna Birnbaum, Sage Aviles, and Meera Sundaram

NOTE: The reviews and decision letters are unedited and appear as submitted by the reviewers.
In extremely rare instances and as determined by a Senior Editor or the EIC, portions of a review may be redacted. If a review is signed, the reviewer has agreed to no longer remain anonymous.
The review history appears in chronological order.

Review Timeline:

Submission Date:	2026-07-18
Editorial Decision:	2026-08-09
Revision Received:	2026-08-14
Accepted:	2026-08-14

August 9, 2026
RE: GENETICS-2026-309741

Dear Meera:

I am pleased to accept your manuscript titled "A Lipocalin and a Hedgehog-related protein are partners in the C. elegans pre-cuticle apical extracellular matrix" for publication in GENETICS, pending minor revision. The reviewers and I are enthusiastic about your work.

The reviewers provided a few suggestions for you to consider. Please submit your revision along with a very brief description of how you modified the manuscript in response to the reviewers' suggestions (which can be viewed at the bottom of this email). I expect you should be able to submit a revised manuscript within ~10 days. A suitably revised manuscript will be acceptable for publication; I don't expect to send it out for review.

Prior to uploading your revised manuscript please format it according to GENETICS style and ensure you have all required elements. Author Guidelines are at https://academic.oup.com/genetics/pages/author-guidelines#section-12. These guidelines have been updated with new requirements and your careful attention is required to avoid delays at the source file stage post-acceptance.

When revising the ms., please make an effort to shorten it, because that almost always improves a manuscript. We urge authors to heed the advice of Strunk and White: "omit needless words"1. Follow this link to submit the revised manuscript: Link Not Available

Thank you for submitting this story to Genetics.

Sincerely,

David

David Greenstein
Senior Editor
GENETICS

Approved by:
Howard Lipshitz
Editor in Chief
GENETICS

Reviewer comments:
Reviewer #1 :

Summary:

This work by Serra et al shows that the C. elegans proteins LPR-3 and WRT-10 interact in the pre-cuticle and act together to pattern the lateral cuticle alae. During C. elegans cuticle formation, collagen proteins are deposited in patterns imposed by a transient extracellular matrix pre-cuticle, and this patterning allows for specialized cuticle substructures such as the annuli, the lateral alae, and the vulva. In this study, the authors show that LPR-3 and WRT are apical extracellular matrix proteins that colocalize in the L4 larval pre-cuticle. Using Alphafold3 to model LPR-3 and WRT-10, the authors show that these proteins may interact directly via a small N-terminal sequence in LPR-3. To test this possibility, they created a gene edit of lpr-3 deleting 7 amino acids of LPR-3 predicted to bind WRT-10. They show that deletion of either WRT-10 alone, or of the 7 interacting amino acids in LPR-3, cause similar patterning defects in the lateral alae. They also use elegant microscopy to show that loss of the 7-amino acid LPR-3 region does not alter LPR-3 pre-cuticle pattern, but causes widespread loss and disruption of the WRT-10 protein. Based on these data, they propose a model that the pre-cuticle alae, annuli, and vulva patterns are composed of at least 3 apical extracellular matrix proteins: a still unknown "landing pad," which in turn recruits LPR-3, which in turn recruits WRT-10.

Strengths:

1. The work is of high importance to specialists studying the apical extracellular matrix. Although the LPR-3 and WRT-10 are not well-conserved beyond nematodes, these are members of Lipocalin and Hedgehog protein families, which are found throughout animals. Although it is beyond the scope of this work to test directly, the new insights here may suggest potential roles for similar proteins in the vertebrate extracellular matrix.
2. The work presents compelling and straightforward evidence, experimental design, and data analysis. For example, I would highlight Figure 2 as an exemplary presentation of Alphafold protein modeling. Although compact, it contains all information that a reader requires - the visual models, confidence scores, protein alignments, and conservation. The manuscript is uncommonly well-written.
3. The work describes new insights supporting a "Landing Pad" model of extracellular pre-cuticle and cuticle patterning. This model posits that specific protein-protein interactions allow for broadly secreted extracellular proteins to be recruited and incorporated in patterned substructures. This work describes one such interaction, between the N-terminal beta strand of LPR-3 and WRT-10.

Weaknesses:

None noted.

Minor comments:

1. Alphafold3 modeling predicted that interaction of LPR-3 and WRT-10 induced a secondary structure change, allowing a region of the LPR-3 N-terminus to form a beta strand. The authors refer to this change as a beta strand addition or removal." (E.g. in the heading "Alphafold3 predicts that WRT-10 and LPR-3 can physically interact via a b-strand addition mechanism.") I would suggest adding a statement defining the words addition or removal as changes in protein conformation, rather than other alternatives that readers may associate with those words, such as protein modification or cleavage.

Reviewer #2 :

Apical extracellular matrices are critical structures that cover and protect exposed surfaces of the body. How these complex structures are built is still poorly understood. In Caenorhabditis elegans, the cuticle functions as an exoskeleton and undergoes precise patterning at each larval stage. Essential to the cuticle pattern is the transient expression of pre-cuticle proteins. The mechanisms by which pre-cuticle proteins assemble is the subject of ongoing work. In this manuscript, Serra et al. convincingly demonstrate that two apical extracellular matrix proteins, WRT-10 and LPR-3, physically interact as part of pre-cuticle patterning. Furthermore, the details of this interaction are consistent with a "landing pad" model, whereby the presence of particular proteins provides an anchor for additional substrates as the apical extracellular matrix is built.

To summarize the data presented, the authors start by demonstrating that endogenously tagged alleles of WRT-10 and LPR-3 colocalize, and Alphafold-3 predicts that the proteins physically interact. To test this prediction, the authors delete a small region of the LPR-3 N-terminus, predicted to be the site of interaction. Careful analysis strongly supports the authors' prediction. First, the N-terminal deletion caused alae defects similar to those seen in wrt-10 mutants, and removing wrt-10 from the deletion mutant did not enhance these defects. Second, LPR-3 patterning within the developing alae is disrupted similarly when the N-terminal fragment is deleted and when wrt-10 is removed. Finally, they find that WRT-10 patterning is disrupted across multiple tissues when the N-terminal fragment of LPR-3 is deleted, while LPR-3 is patterned normally both when the N-terminal fragment is deleted and also in a wrt-19(0) background. This latter point is consistent with the landing pad model.

This paper is extremely clear, thorough, and convincing. I have only one "major" comment that is editorial but nonetheless important. The other minor comments are small points for the authors to consider, but are fully optional from my perspective. I leave it to their judgement.

Major comment:
1. Please ensure that all figures are colorblind friendly. In particular, Figure 3C seems like it might be difficult for some, since there is a great deal of green and red in that figure.

Minor comments/suggestions:
1. Consider whether it might be useful to show Alphafold predictions of the tagged proteins, to bolster the interpretation of their use for visualization.
2. Consider rewording the sentence "Colored bars indicate method for quantifying fluorescence intensity in B,D." (Figure 4 legend). It took me a few readings of this statement, in combination with other explanations later in the figure legend and in the methods, to understand that the "method" referred to the areas where measurements were taken for quantification.
3. Consider whether Figure S2 should be a main figure. I understand putting these data in the supplement, since they are essentially negative data, but the negative data still make an important point.
4. I wondered whether the Brief Investigation format would work better for this paper, since the paper is focused around one argument, nicely bolstered by a great deal of data. However, I do not think that the paper should be trimmed as it is already

concise, and all of the information provided is relevant. If changing the format would lead to cuts in the text or figures, then it's better to stay as is. This would be a decision between the authors and the editor.

RE: GENETICS-2026-309741

Reviewer #1:

Minor comments:

1. Alphafold3 modeling predicted that interaction of LPR-3 and WRT-10 induced a secondary structure change, allowing a region of the LPR-3 N-terminus to form a beta strand. The authors refer to this change as a beta strand addition or removal." (E.g. in the heading "Alphafold3 predicts that WRT-10 and LPR-3 can physically interact via a b-strand addition mechanism.") I would suggest adding a statement defining the words addition or removal as changes in protein conformation, rather than other alternatives that readers may associate with those words, such as protein modification or cleavage.

We added this clarification to two places in the manuscript:

Results p. 10. Alphafold3 predicted an interaction between the WRT-10 WRT domain and a small region of the extreme LPR-3 N-terminus, which changed conformation to form an extra β-strand specifically in the presence of WRT-10 (Fig. 2b).

Discussion p. 15. In the interaction model described here, an initially unstructured region of the LPR-3 N-terminus changes conformation to form an extra β-strand that incorporates into the WRT-10 β-sandwich structure, without removal of any of the pre-existing WRT-10 β-strands.

Reviewer #2:

Major comment:
1. Please ensure that all figures are colorblind friendly. In particular, Figure 3C seems like it might be difficult for some, since there is a great deal of green and red in that figure.

We changed the color scheme in Figure 3C and adjusted the color opacity in other figures to be more accessible.

Minor comments/suggestions:
1. Consider whether it might be useful to show Alphafold predictions of the tagged proteins, to bolster the interpretation of their use for visualization.

We added Alphafold3 scores for tagged proteins to Table S2 and added a new supplementary figure (Figure S2) showing the Alphafold3 model for interaction between mCherry::LPR-3 and WRT-10 compared to mCherry::LPR-3(ΔNter) and WRT-10. The text (pp. 11-12) now cites these to justify our approach of using the mCh::LPR-3 fusion for making the deletion mutant.

2. Consider rewording the sentence "Colored bars indicate method for quantifying fluorescence

intensity in B,D." (Figure 4 legend). It took me a few readings of this statement, in combination with other explanations later in the figure legend and in the methods, to understand that the "method" referred to the areas where measurements were taken for quantification.

This was re-worded to say: Colored bars represent regions where line scans were taken to quantify peak fluorescence intensity.

3. Consider whether Figure S2 should be a main figure. I understand putting these data in the supplement, since they are essentially negative data, but the negative data still make an important point.

We agreed with this suggestion. The original Figure S2 is now main Figure 5.

4. I wondered whether the Brief Investigation format would work better for this paper, since the paper is focused around one argument, nicely bolstered by a great deal of data. However, I do not think that the paper should be trimmed as it is already concise, and all of the information provided is relevant. If changing the format would lead to cuts in the text or figures, then it's better to stay as is. This would be a decision between the authors and the editor.

We stuck with the Investigation format since the text length and number of figures is too high for a Brief Investigation.

August 14, 2026
RE: GENETICS-2026-309741R1

Dr. Meera V. Sundaram
University of Pennsylvania Perelman School of Medicine
Genetics
Univ. of Pennsylvania School of Medicine, Dept. of Genetics, 446a Clinical Research Building, 415 Curie Blvd.
Philadelphia, Pennsylvania 19104

Dear Meera:

Congratulations, your manuscript titled "A Lipocalin and a Hedgehog-related protein are partners in the C. elegans pre-cuticle apical extracellular matrix" is accepted for publication in GENETICS! Many thanks for submitting your research to the journal.

To Proceed to Publication:
1. Format your article according to GENETICS style: https://academic.oup.com/genetics/pages/author-guidelines

2. Ensure that you comply with data and community resource citation guidelines:
https://academic.oup.com/genetics/pages/author-guidelines#section-5-9-2

3. Upload your final files at https://genetics.msubmit.net

4. Add oupsupport@scipris.com and genetics.oup@novatechset.com (or the domains @scipris.com and @novatechset.com) to your email program's "safe senders" list. You will be contacted by both at various points during the production process.

Notes:
- Your currently-accepted manuscript (unedited, as submitted, reviewed, and accepted) will be published at GENETICS and deposited into PubMed as an Advance Access article. Notify sourcefiles@thegsajournals.org before signing your license if you do not wish to publish your article via Advance Access.

- We invite you to submit an original color figure related to your paper for consideration as cover art. Please note that submissions created with AI image generation tools are not eligible. Please email your submission to the editorial office or upload it with your final files. You can submit a small-sized image for evaluation, and if selected, the final image must be a TIFF file 2513px wide by 3263px high (8.375 by 10.875 inches; resolution of 600ppi). Please avoid graphs and small type.

- After files are sent to Oxford University Press we use SciPris to manage article licensing and payment. If you do not have a SciPris account, you will receive an email from no-reply@scipris.com to sign up to use Oxford University Press' author portal. After logging in, follow the online instructions to sign your license and arrange any payment due.

If you have any questions or encounter any problems while uploading your accepted manuscript files, please email the editorial office at sourcefiles@thegsajournals.org.

Sincerely,

David

David Greenstein
Senior Editor
GENETICS

Approved by:
Howard Lipshitz
Editor in Chief
GENETICS
