# Supplementary material for: A lipocalin and a Hedgehog-related protein are partners in the Caenorhabditis elegans precuticle apical extracellular matrix
Source: Genetics. 2026 Aug 19;234(3):iyag218. doi: 10.1093/genetics/iyag218 (PMC13732703; doi:10.1093/genetics/iyag218)
Supplement: Supplementary file 1 [file iyag218_supplementary_data.zip › Figure_S1_GENETICS-2026-309741.pdf]

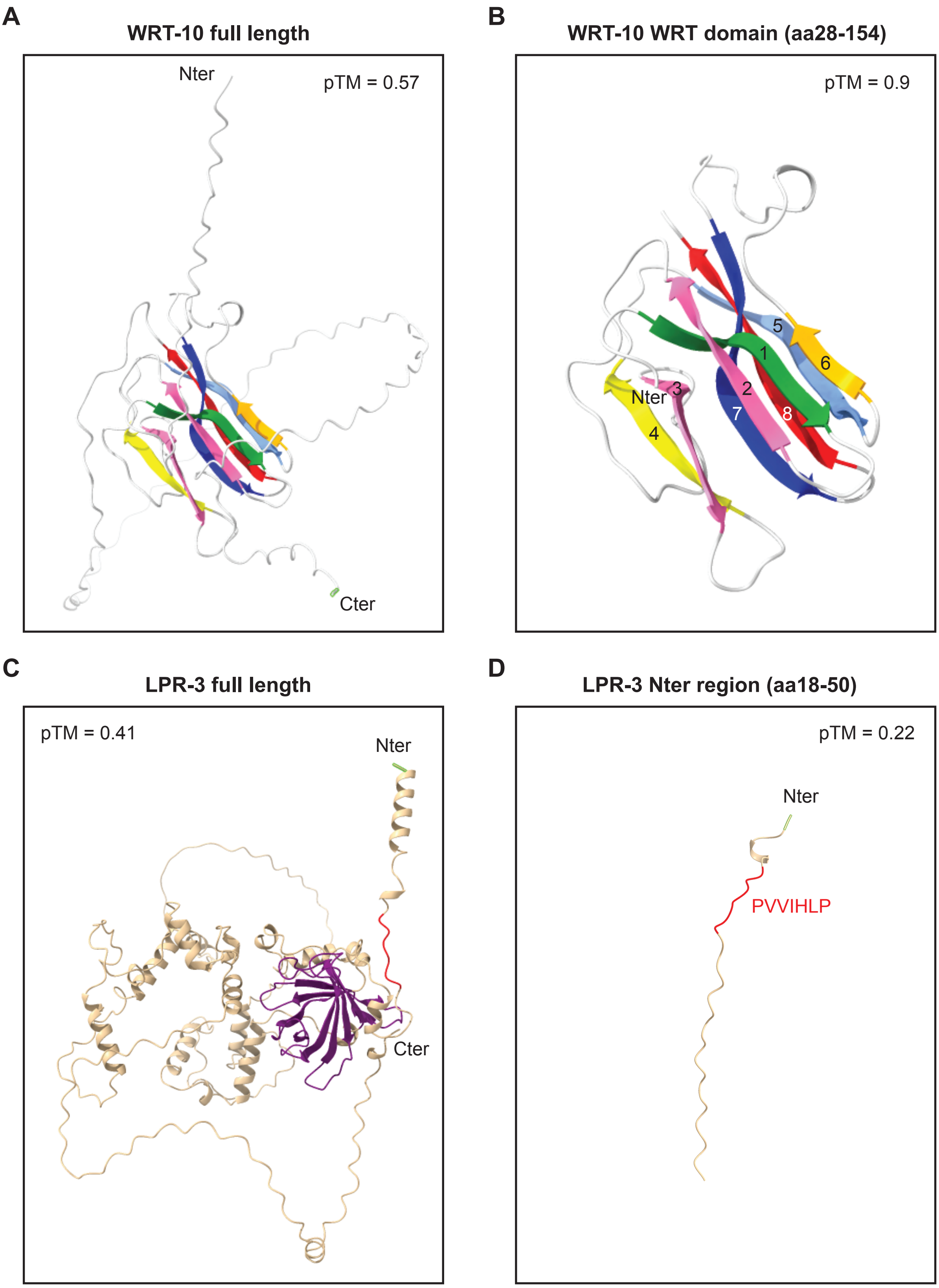
A
WRT-10 full length
Nter
pTM = 0.57
Cter
B
WRT-10 WRT domain (aa28-154)
pTM = 0.9
5
1
6
Nter
3
2
7
8
4
C
LPR-3 full length
pTM = 0.41
Nter
Cter
D
LPR-3 Nter region (aa18-50)
pTM = 0.22
Nter
PVVIHLP
