# Supplementary material for: A lipocalin and a Hedgehog-related protein are partners in the Caenorhabditis elegans precuticle apical extracellular matrix
Source: Genetics. 2026 Aug 19;234(3):iyag218. doi: 10.1093/genetics/iyag218 (PMC13732703; doi:10.1093/genetics/iyag218)
Supplement: Supplementary file 1 [file iyag218_supplementary_data.zip › Figure_S2_GENETICS-2026-309741.pdf]

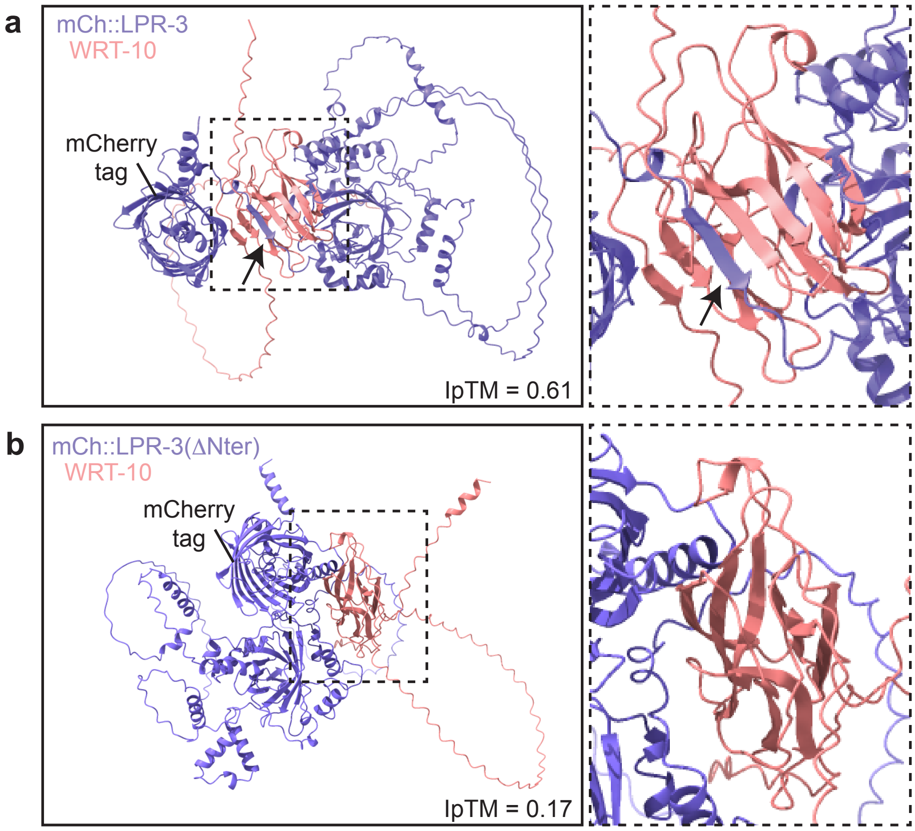
a
mCh::LPR-3
WRT-10
mCherry
tag
IpTM = 0.61
b
mCh::LPR-3(ΔNter)
WRT-10
mCherry
tag
IpTM = 0.17
