# Supplementary material for: A lipocalin and a Hedgehog-related protein are partners in the Caenorhabditis elegans precuticle apical extracellular matrix
Source: Genetics. 2026 Aug 19;234(3):iyag218. doi: 10.1093/genetics/iyag218 (PMC13732703; doi:10.1093/genetics/iyag218)
Supplement: Supplementary file 1 [file iyag218_supplementary_data.zip › Figure_S3_GENETICS-2026-309741.pdf]

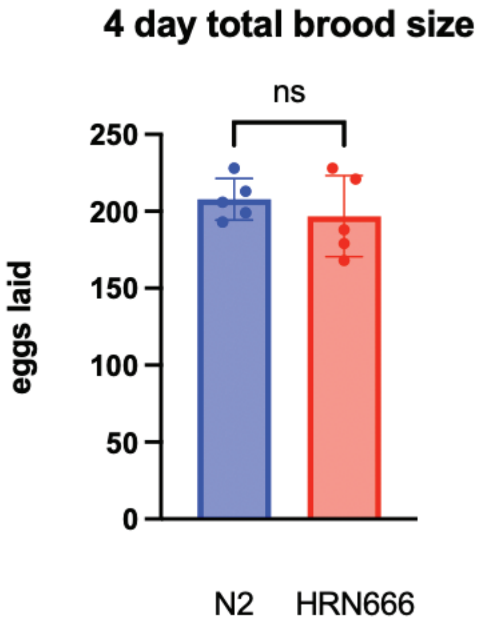
4 day total brood size
ns
eggs laid
250
200
150
100
50
0
N2
HRN666
