# Supplementary material for: A lipocalin and a Hedgehog-related protein are partners in the Caenorhabditis elegans precuticle apical extracellular matrix
Source: Genetics. 2026 Aug 19;234(3):iyag218. doi: 10.1093/genetics/iyag218 (PMC13732703; doi:10.1093/genetics/iyag218)
Supplement: Supplementary file 1 [file iyag218_supplementary_data.zip › Supplementary_Material_Legends_GENETICS-2026-309741.docx]

**Supplemental Information**

**Table S1.** *C. elegans* strains used in this study.

**Table S2.** Alphafold3 pairwise test results of *C. elegans* lipocalins and WRT domain proteins.

**Table S3.** Source data for Fig. 3C.

**Table S4.** Source data for Fig. 4B.

**Table S5.** Source data for Fig. 4D.

**Table S6.** Source data for Fig. 5C.

**Table S7.** Source data for Fig. 5F.

**Table S8.** Source data for Fig. 6C.

**Table S9.** Source data for Fig. 6E.

**Fig. S1. Alphafold3 predicted structures for WRT-10 and LPR-3.**

a) Full-length WRT-10. b) WRT-10 WRT domain (amino acids 28-154). Predicted β-strands are colored and numbered beginning at the most N-terminal region. **c**) Full length LPR-3. d) LPR-3 N-terminal region (amino acids 18-50). The region predicted to bind WRT-10 (PVVIHLP) is indicated in red. Alphafold3 pTM scores are indicated.

**Fig. S2. Alphafold3 predicted structures for WRT-10 interacting with tagged mCh::LPR-3**

mCh::LPR-3 predicted structures are shown in dark purple and WRT-10 structures are shown in light pink. Boxed regions in the left images are shown at higher magnifciation on the right. a) The N-terminal tag on LPR-3 did not appear to interfere with the predicted interaction, and the iPTM score (0.61) was similar to that of the untagged proteins (Fig. 2 and Table S2). b) Deletion of the LPR-3 Nter motif greatly reduced the iPTM (0.17) and eliminated the β-strand addition in the interaction model.

**Fig. S3. *wrt-10(-)* mutants have normal brood size.**

Fertilized eggs released by five N2 (WT) and five HRN666 (*wrt-10(aus36))* self-fertilizing hermaphrodites were monitored from lay initiation to cessation on day 4. ns, no significant difference, Mann-Whitney U test. Note that this experiment was not powered to detect subtle changes, nor did we assess broods from mated animals.
